# Enhanced performance of nitride-based ultraviolet vertical-injection light-emitting diodes by non-insulation current blocking layer and textured surface

**DOI:** 10.1186/1556-276X-9-699

**Published:** 2014-12-29

**Authors:** Yen Chih Chiang, Bing Cheng Lin, Kuo Ju Chen, Chien Chung Lin, Po Tsung Lee, Hao Chung Kuo

**Affiliations:** Institute of Lighting and Energy Photonics, National Chiao Tung University, No.301, Gaofa 3rd Rd., Guiren Dist., Tainan City, 71150 Taiwan; Department of Photonics and Institute of Electro-Optical Engineering, National Chiao Tung University, 1001 University Road, Hsinchu, 300 Taiwan; Institute of Photonics System, National Chiao Tung University, No.301, Gaofa 3rd Rd., Guiren Dist., Tainan City, 71150 Taiwan

**Keywords:** Gallium nitride, Light-emitting diode, Vertical injection, Ultraviolet, Current blocking layer, Textured surface

## Abstract

For the purpose of light extraction and efficiency enhancement, the nitride-based ultraviolet vertical-injection light-emitting diodes (UV-VLEDs) with non-insulation current blocking layer (n-CBL) and optimized textured surface were fabricated. The optical and electrical characteristics were investigated in this n-CBL UV-VLED. Furthermore, the efficiency of optimized structure was improved by 5 ~ 6 times compared to our reference.

## Background

The importance of high-power nitride-based light-emitting diodes (LEDs) has been rising since the last 20 years, and they are extensively used in outdoor displays, vehicle lightings, and backlights. With current trends in the consumer market, they are on the pace to replace incandescent bulbs and fluorescent lamps in the next decade [1]. While blue LEDs take the biggest part of GaN-based devices, ultraviolet (UV) emitters are also very crucial for chemical ink curing, flame detection, optical storage, water purification, and phosphor excitation [2]. In the mean time, the high-quality blue/green LEDs are commercially available, but the UV-LEDs are still low in efficiency and difficult to manufacture. Many reasons contribute to this situation: one of them is due to the inherent absorption from the GaN layer. Another one is caused by bad thermal conduction of the sapphire substrate. Third, low light extraction due to total internal reflection also plays a certain role [3]. Finally, the notorious efficiency droop that exhibits at high current condition can also deteriorate the performance of UV-LEDs [4, 5].

The reduction of efficiency is a direct loss of output power and thus leads to the increase of cost. How to solve this droop issue and improve external quantum efficiency (EQE) become important for both industrial companies and academic labs, and many previous efforts have been demonstrated [6–10]. Other than the fundamental droop issue, recently, laser lift-off (LLO) LEDs were demonstrated to eliminate the bad thermal dissipation of sapphire. Some scholars are using very high thermal conductivity material to solve this problem, such as electroplated copper (400 W/k-m) or metal base substrate [11, 12]. Because of difficulties in metal cutting, silicon substrate becomes an alternative to replace sapphire substrate as well [13–15]. Great enhancements at high current efficiency and output power via wafer bonding technology and LLO have been shown [16–18]. Other non-epitaxial improvements, such as dielectric current blocking layer (CBL) and surface textures, can also provide significant results in the past [19–30].

In this article, different technologies such as a non-insulation current blocking layer, wafer bonding, LLO, and surface treatment processes were implemented to fabricate the nitride-based ultraviolet vertical-injection light-emitting diodes (UV-VLEDs). The optical and electrical property enhancement of UV-VLEDs will be reported in the following.

## Methods

The LED structures were grown on (0001) sapphire substrates by a metal-organic chemical vapor deposition (MOCVD) system. The epitaxial structure of the 365-nm UV-VLED is composed of a 2.2-μm-thick Si-doped n-GaN layer, a 2.0-μm-thick Si-doped n-AlGaN cladding layer, six-period AlGaN/InGaN multiple quantum wells (MQWs), a 0.2-μm-thick Mg-doped p-AlGaN electron blocking layer, and a 0.3-μm-thick Mg-doped p-GaN layer. A 20-nm non-insulating current blocking layer (n-CBL) can be patterned on the top of the p-GaN surface after the growth. This n-CBL layer can be used as a current aperture layer, which regulates the current flow via different resistivities among different current paths [31, 32].

**Figure 1:**
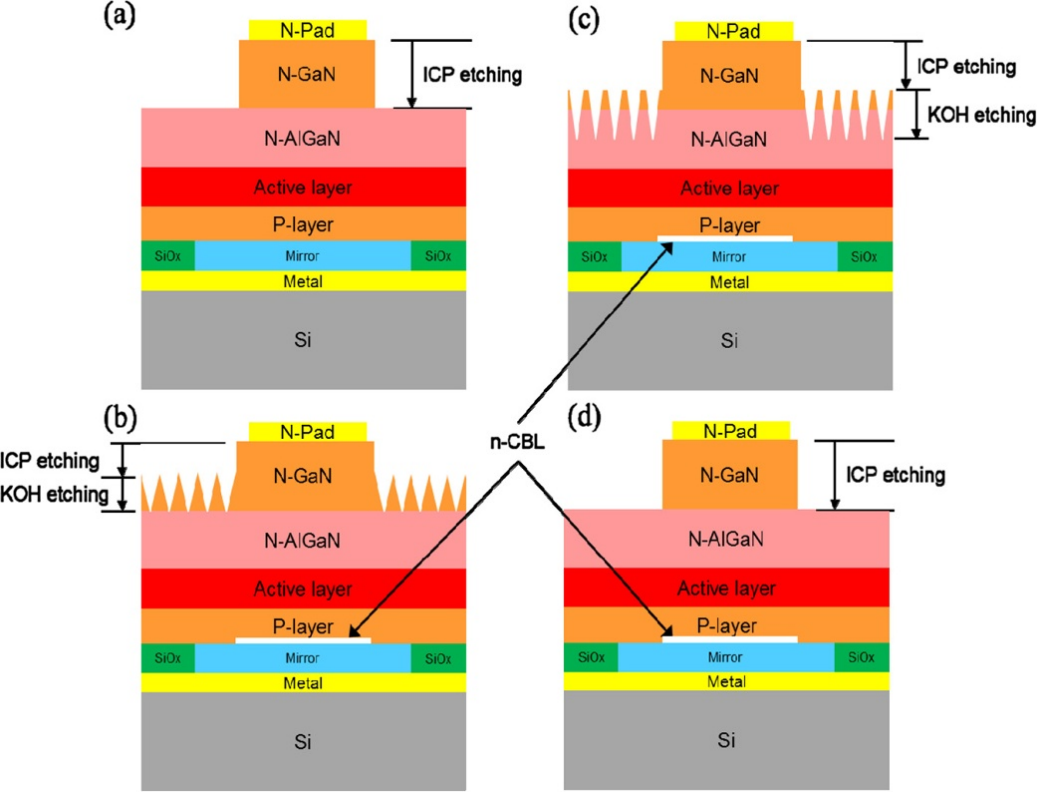
**Schematic diagram of UV-VLEDs.** Schematic diagram of **(a)** C-VLED, **(b)** UV-VLED with n-CBL and larger pyramid textured surface (UV-VLED-1), **(c)** UV-VLED with n-CBL and smaller pyramid textured surface (UV-VLED-2), and **(d)** UV-VLED only with n-CBL (UV-VLED-3).

Regular semiconductor processes are used to fabricate the devices. The multilayer metal systems Ni(5 Å)/Ag(1,000 Å)/Ti(300 Å)/Pt(500 Å)/Ti(300 Å)/Pt(500 Å) and Cr(300 Å)/Pt(500 Å)/Au(12,000 Å) were deposited by electron beam evaporation at a pressure of 1 × 10^-6^ Torr to serve as the p-contact and bonding metal. After the metal deposition, the specimen was bonded to the Si substrate with Cr(300 Å)/Pt(500 Å)/Au(12,000 Å) at 220°C for 30 min. Through a wafer bonding technique, the substrate is transferred into a highly thermal conductive silicon substrate to provide great thermal dissipation, and this new substrate can potentially provide a platform for light-emitting devices to achieve high brightness operation. Then, the sapphire substrate was removed by an LLO process. A KrF excimer laser at a wavelength of 248 nm with a pulse width of 25 ns was used for the LLO process. The laser with a beam size of 0.3 mm × 0.3 mm was incident from the backside of the substrate onto the sapphire/n-GaN interface to decompose GaN into Ga and N. After the sapphire substrate removal, the specimen was dipped into a HCl solution to get rid of the residual Ga on the n-GaN. The details of the LLO process are described in [16]. To eliminate the possible UV absorption caused by laser damage in the n-GaN target layer, this layer was removed by inductively coupled plasma (ICP) dry etching. Additionally, in order to enhance light extraction, a 40% KOH solution at 90°C was used to create the surface roughness of the n-GaN epilayer under different time durations: (a) 1 min and (b) 2 min. As shown in Figure 1b,c, a multiple-layer structure of Ti(300 Å)/Al(1,500 Å)/Ni(1,000 Å)/Au(1.2 μm) was deposited on the surface of the n-GaN epilayer to serve as the n-contact. Finally, the UV-VLED chip was cut into square pieces with a dimension of 1.15 mm × 1.15 mm. In addition, a similar UV-VLED structure without the n-CBL and textured surface was also fabricated for comparison, denoted as conventional vertical LEDs (C-VLED). Note that the depth of the etched n-GaN of C-VLED is 2.2 μm. Figure 1 shows the schematic diagram of these UV-VLEDs.

**Figure 2:**
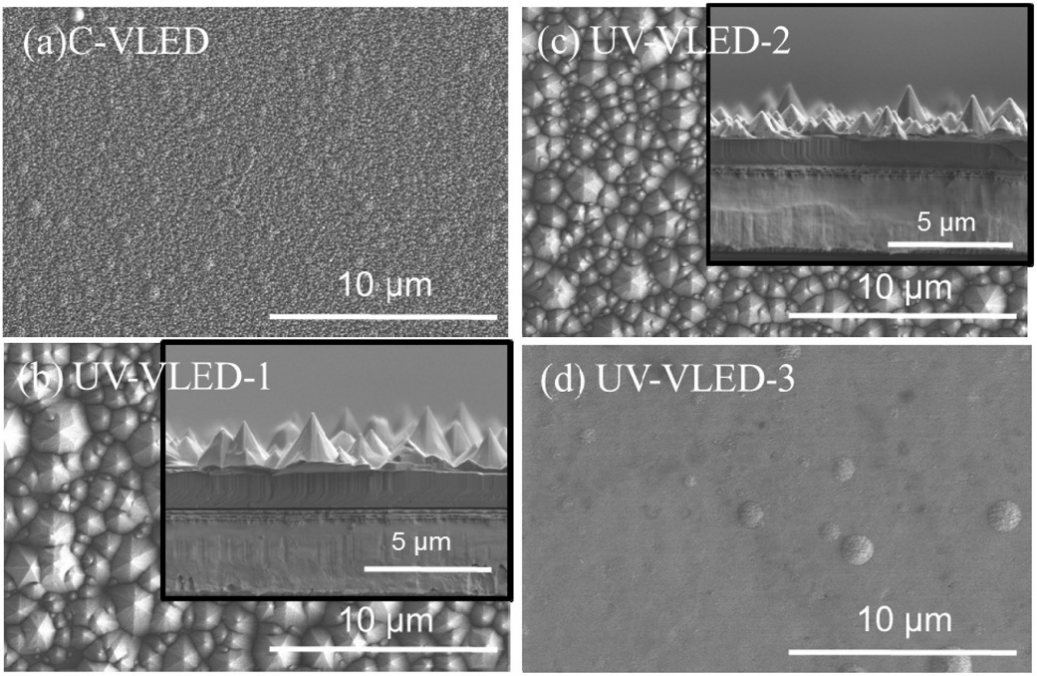
**SEM images of emission area. (a)** C-VLED after ICP 22-kÅ-deep dry etching, **(b)** UV-VLED-1 after ICP 5-kÅ-deep dry etching and KOH dipped for 120 s, **(c)** UV-VLED-2 after ICP 15-kÅ-deep dry etching and KOH dipped for 60 s, and **(d)** UV-VLED-3 after ICP 22-kÅ-deep dry etching.

**Figure 3:**
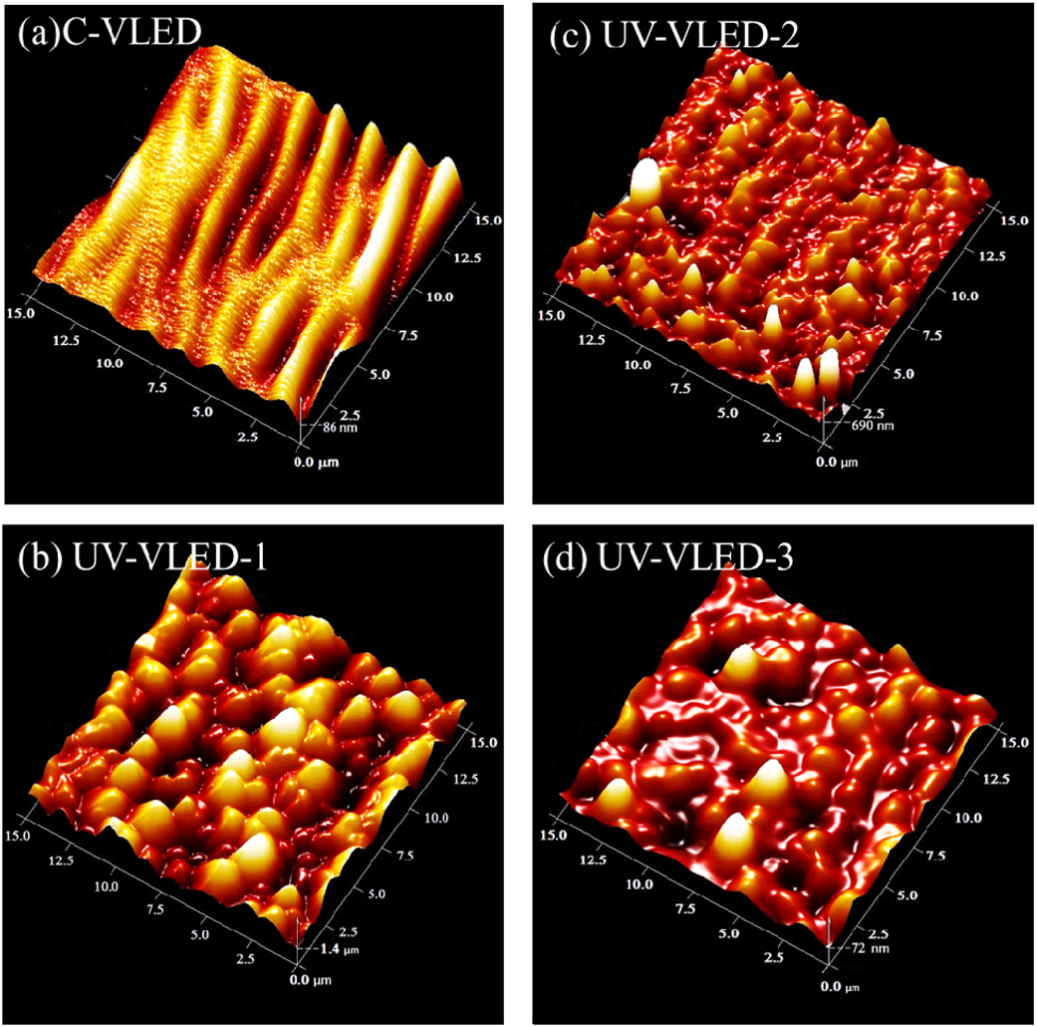
**AFM images of emission area. (a)** C-VLED after ICP 22-kÅ-deep dry etching, **(b)** UV-VLED-1 after ICP 5-kÅ-deep dry etching and KOH dipped for 120 s, **(c)** UV-VLED-2 after ICP 15-kÅ-deep dry etching and KOH dipped for 60 s, and **(d)** UV-VLED-3 after ICP 22-kÅ-deep dry etching.

Figures 2 and 3 present typical scanning electron microscope (SEM) and atomic force microscope (AFM) images of the etched n-GaN surface appearance for these UV-VLED samples. The insets in Figure 2 are the cross-sectional views of the etched surfaces. Among the n-CBL samples, different degrees of surface roughness are also fabricated to test their effects on output power. By varying the KOH etching time, three different degrees of surface morphology and surface roughness (Rms) can be achieved, as shown in Table 1. We noticed that the pyramid dimensions and pyramidal distribution density is inversely proportional with the etching time. With this result, we will find the relationship of luminous intensity and efficiency corresponds to different degrees of roughness.

**Table 1:** **Pyramid size and surface roughness in each UV-VLED sample**

Type of device	Pyramid size	Surface roughness (Rms)
UV-VLED-1	0.8 to 1.5 μm	377 nm
UV-VLED-2	0.3 to 1.0 μm	163 nm
UV-VLED-3	Flat	17 nm

## Results and discussion

**Figure 4:**
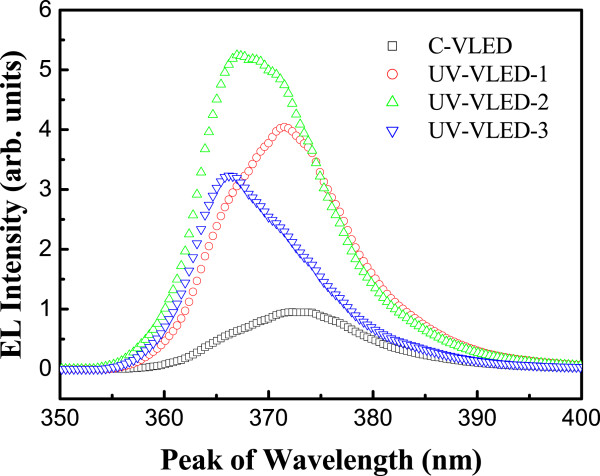
**Room-temperature EL spectra of UV-VLEDs at 350 mA.**

**Figure 5:**
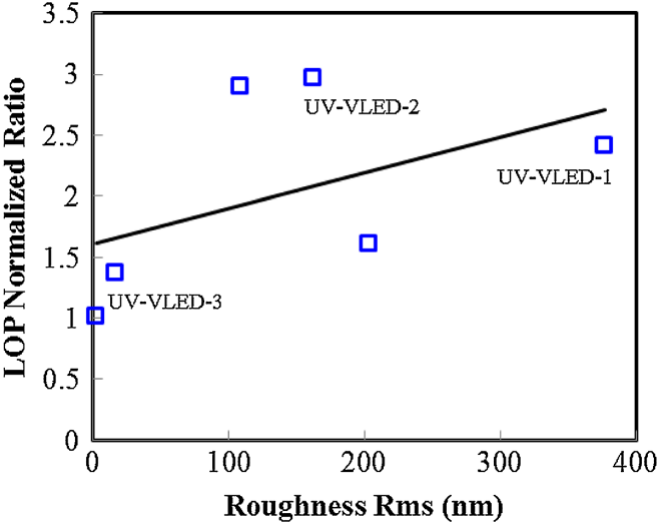
**Light output power corresponds to different roughness Rms conditions.**

Figure 4 shows the room-temperature electroluminescence (EL) spectra of these UV-VLEDs under a forward injection current of 350 mA. The luminescent properties of the fabricated UV-VLEDs were measured by a calibrated integrating sphere at room temperature. The emission dominant wavelength for these UV-VLEDs was about 366 ~ 371 nm. As shown in Figure 4, UV-VLED-2 displays superior emission intensity. In addition to discuss changes in intensity, we also observed full width at half maximum (FWHM) and wavelength shift problem. The FWHM of spectrum in these samples is as follows: C-VLED (about 14.8 nm), UV-VLED-1 (about 14.4 nm), UV-VLED-2 (about 13.3 nm), and UV-VLED-3 (about 12.5 nm). From the EL results, the dominant mechanisms for the wavelength shifts and spectral width changes can be attributed to two major reasons: (1) The different stresses on the epitaxial structure due to the thinned GaN substrate. This change of stress can relieve some of the quantum-confined Stark effect (QCSE) on the MQW, which can move the EL peak to shorter wavelength and narrower linewidth [33–36]. (2) With better current spreading or reduced current crowding effect, the local heating of the chip can be greatly improved and such a longer wavelength can be observed in the C-LED, which does not have an n-CBL layer [37–39].In Figure 5, the correlation between the surface morphology and output power is presented. A positive correlation could be observed from the plot. The rougher the surface is, the higher the output power of the device becomes, which is a clear indication of better light extraction. Following this result, we pick some marked devices to execute a detailed optical and electrical characteristic comparison.

**Figure 6:**
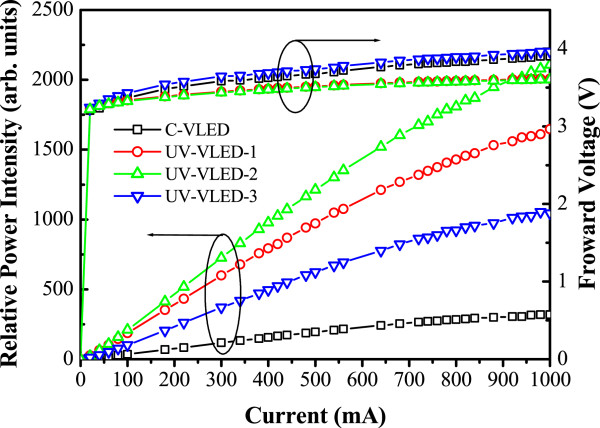
**Light-current-voltage (** ***L*** **-** ***I*** **-** ***V*** **) characteristics of UV-VLEDs.**

**Figure 7:**
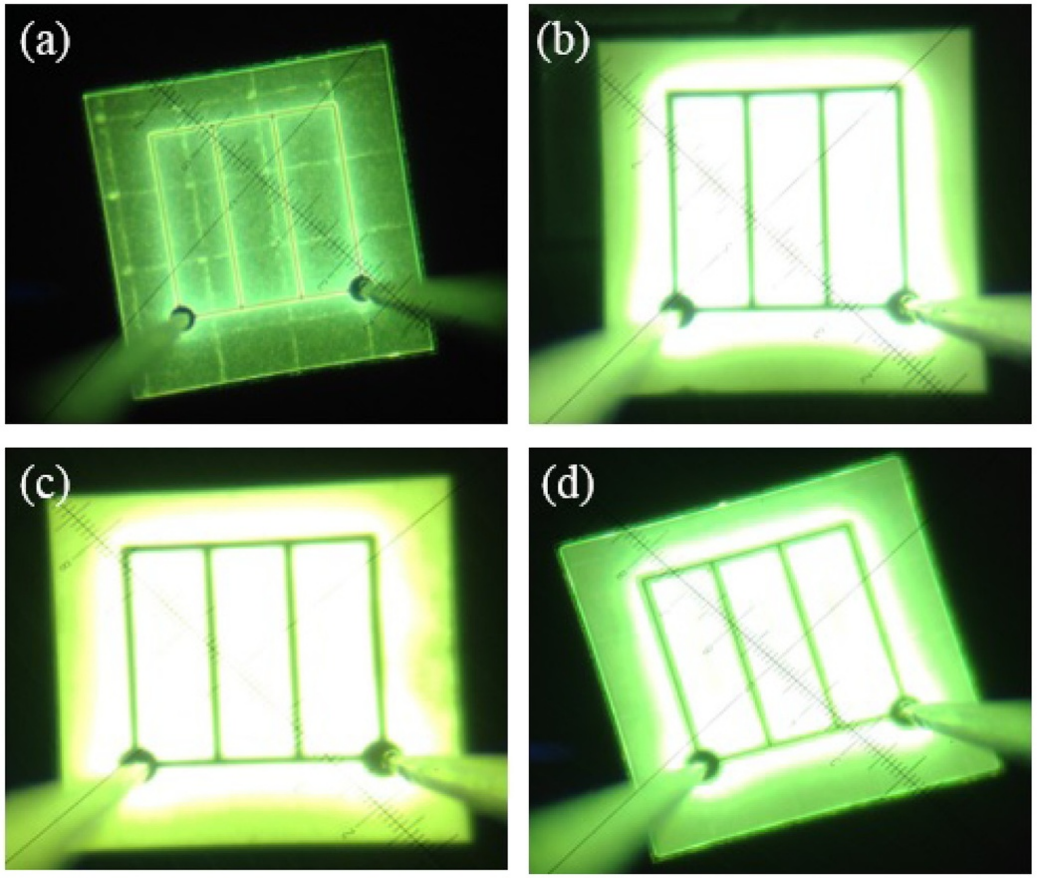
**Near-field images of UV-VLEDs. (a)** C-VLED, **(b)** UV-VLED with n-CBL and larger pyramid textured surface (UV-VLED-1), **(c)** UV-VLED with n-CBL and smaller pyramid textured surface (UV-VLED-2), and **(d)** UV-VLED only with n-CBL (UV-VLED-3).

Figure 6 illustrates the light-current-voltage (*L*-*I*-*V*) characteristics of these UV-VLEDs. With an injection current of 350 mA, the forward voltages were about 3.3 ~ 3.6 V for these UV-VLEDs. However, the forward voltage of UV-VLED-2 is decreased by 0.15 V from the value of C-VLED. We believe the reason is due to the damages from various etching processes. Great enhancement in output power can be observed from the UV-VLED-1, UV-VLED-2, and UV-VLED-3 when compared to the C-VLED result. Among these three cases, the UV-VLED-2 posts the best increment, and this great output power improvement (approximately 522%) could be mainly attributed to the n-CBL and textured surface. From cross-examination among our samples, different enhancement mechanisms can be identified comparatively. Assuming the epitaxial qualities of these samples are the same, the different fabrication processes distinguished the performances of the chips. First, between the C-VLED and UV-VLED-3, the only difference is the insertion of the n-CBL layer, and the power enhancement is 214% at 350 mA. Second, among the three samples of UV-VLED-1, UV-VLED-2, and UV-VLED-3, the only difference among them is the surface texture and thus the light extraction efficiency. The *L*-*I* comparison shows 98% and 61% of increase between UV-VLED-1 vs. UV-VLED-3 and between UV-VLED-2 vs. UV-VLED-3, respectively. Combining these two effects (n-CBL and texture), the overall enhancement factor is calculated as [(1 + 98%) × (1 + 214%) - 1] = 521%, which is close to the observed value between UV-VLED-2 and C-VLED (522%). The near-field images of these UV-VLEDs are shown in Figure 7. It can be seen that the light emission is more uniform in the UV-VLED-2 than that of the C-VLED. Between C-VLED and UV-VLED-3, the uniformity improves greatly due to the introduction of the n-CBL which can distribute current more evenly and avoid current crowding.

**Figure 8:**
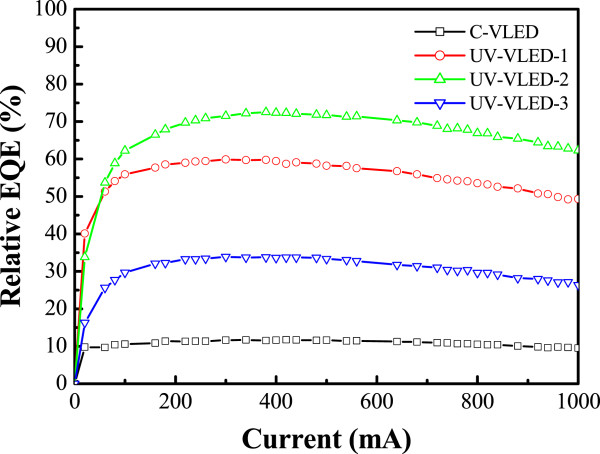
**Relative EQE curves of UV-VLEDs.**

Figure 8 shows the relative EQE as a function of current for these UV-VLEDs, measured under room temperature pulse mode operation. A significant difference in device efficiency was observed at an injection current of 350 mA. The efficiency values of the C-VLED, UV-VLED-1, UV-VLED-2, and UV-VLED-3 were 11%, 60%, 72%, and 34%, respectively. Compared with the C-VLED, the efficiency of UV-VLED-2 was therefore increased by 6.5 times. The details of comparison data are shown in Tables 2, 3, and 4. This influence from adopted n-CBL and various textured surfaces was as below: Comparing with or without n-CBL, the efficiency of the UV-VLEDs was also increased by 3.1 times. In addition, the efficiencies of various textured surfaces were increased by 1.8 and 2.1 times, respectively.

**Table 2:** **Key parameters and measured data in different sample conditions**

	Sample ID	Adopted n-CBL	GaN removed depth (kÅ)	KOH etching time (min)	Rms (nm)	LOP	EQE (%)
(a)	C-VLED	No	22	0	20	138	11
(b)	UV-VLED-1	Yes	5	2	163	698	60
(c)	UV-VLED-2	Yes	15	1	377	858	72
(d)	UV-VLED-3	Yes	22	0	17	433	34

**Table 3:** **Improvement of light output power correspond to different conditions**

	Sample ID	LOP	LOP improvement		
			n-CBL influence correspond to (a)	Roughness influence correspond to (d)	Influence of combination correspond to (a)
(a)	C-VLED	138			
(b)	UV-VLED-1	698		61%	
(c)	UV-VLED-2	858		98%	522%
(d)	UV-VLED-3	433	214%		

**Table 4:** **Improvement of external quantum efficiency correspond to different conditions**

	Sample ID	EQE (%)	EQE improvement		
			n-CBL influence correspond to (a)	Roughness influence correspond to (d)	Influence of combination correspond to (a)
(a)	C-VLED	11			
(b)	UV-VLED-1	60		1.8	
(c)	UV-VLED-2	72		2.1	6.5
(d)	UV-VLED-3	34	3.1		

## Conclusions

In conclusion, the UV-VLEDs were demonstrated and investigated - including the significance of the existence of n-CBL and discussion on the influence from extents of textured surface. The n-CBL influence is mentioned in the previous text very clearly. Furthermore, the results comprised n-CBL and textured surface; these two processes that indicated the output power intensity and relative external quantum efficiency of the better UV-VLED-2 increased approximately 525% and 6.5 times compared to the C-VLED, respectively. Consequently, we believe that the n-CBL and optimized textured surface should be promising for the future applications of solid-state lighting.

## Abbreviations

AFM: atomic force microscope
CBL: current blocking layer
C-VLED: conventional vertical LEDs
EL: electroluminescence
EQE: external quantum efficiency
FWHM: full width at half maximum
LEDs: light-emitting diodes
*L*-*I*-*V*: light-current-voltage
LLO: laser lift-off
MOCVD: metal-organic chemical vapor deposition
n-CBL: non-insulation current blocking layer
SEM: scanning electron microscope
UV: ultraviolet
UV-VLEDs: ultraviolet vertical-injection light-emitting diodes.
